# EUFOREA consensus on biologics for CRSwNP with or without asthma

**DOI:** 10.1111/all.13875

**Published:** 2019-07-15

**Authors:** Wytske J. Fokkens, Valerie Lund, Claus Bachert, Joaquim Mullol, Leif Bjermer, Jean Bousquet, Giorgio W. Canonica, Lauren Deneyer, Martin Desrosiers, Zuzana Diamant, Joseph Han, Enrico Heffler, Claire Hopkins, Roger Jankowski, Guy Joos, Andrew Knill, Jivianne Lee, Stella E. Lee, Gert Mariën, Benoit Pugin, Brent Senior, Sven F. Seys, Peter W. Hellings

**Affiliations:** ^1^ Department of Otorhinolaryngology Amsterdam University Medical Centres, Location AMC Amsterdam Amsterdam The Netherlands; ^2^ European Forum for Research and Education in Allergy and Airway Diseases (EUFOREA) Brussels Belgium; ^3^ Royal National Throat, Nose and Ear Hospital University College London Hospitals London UK; ^4^ Upper Airways Research Laboratory University of Ghent Gent Belgium; ^5^ Division of ENT Diseases, CLINTEC Karolinska Institute, and Department of ENT Diseases Karolinska University Hospital Stockholm Sweden; ^6^ Department of Otorhinolaryngology, Hospital Clínic Universitat de Barcelona, IDIBAPS, CIBERES Barcelona, Catalonia Spain; ^7^ Department of Respiratory Medicine and Allergology Lund University Lund Sweden; ^8^ Department of Respiratory Disease University Hospital Arnaud de Villeneuve Montpellier France; ^9^ Personalized Medicine, Asthma & Allergy - Humanitas Clinical and Research Center IRCCS Rozzano (MI) Italy; ^10^ Department of Biomedical Science Humanitas University Pieve Emanuele (MI) Italy; ^11^ Division of Otolaryngology‐Head & Neck Surgery University of Montreal Hospital Centre (CHUM) Montreal Quebec Canada; ^12^ Department of Clinical Pharmacy & Pharmacology and Department of General Practice UMCG, and QPS‐NL Groningen The Netherlands; ^13^ Department of Respiratory Medicine, First Faculty of Medicine Charles University and Thomayer Hospital Prague Czech Republic; ^14^ Department of Otolaryngology, Head & Neck Surgery Eastern Virginia Medical School Norfolk Virginia; ^15^ Guy’s and St. Thomas’ NHS Foundation Trust London UK; ^16^ ENT Department University Hospital of Nancy, Brabois-ILM Nancy France; ^17^ Department of Respiratory Medicine Ghent University Hospital Gent Belgium; ^18^ Opuscomms London UK; ^19^ Rhinology & Endoscopic Skull Base Surgery UCLA Department of Head & Neck Surgery Los Angeles California; ^20^ Division of Sinonasal Disorders and Allergy, Department of Otolaryngology—Head & Neck Surgery University of Pittsburgh School of Medicine Pittsburgh Pennsylvania, USA; ^21^ Department of Microbiology, Immunology and Transplantation Allergy and Clinical Immunology Research Group Leuven Belgium; ^22^ Division of Rhinology, Allergy, and Endoscopic Skull Base Surgery University of North Carolina at Chapel Hill Chapel Hill North Carolina; ^23^ Department of Otorhinolaryngology‐Head and Neck Surgery University Hospitals Leuven Leuven Belgium

**Keywords:** asthma, biologics, chronic rhinosinusitis, nasal polyps, type 2 inflammation

## Abstract

Novel therapies such as type 2 targeting biologics are emerging treatment options for patients with chronic inflammatory respiratory diseases, fulfilling the needs of severely uncontrolled patients. The majority of patients with chronic rhinosinusitis with nasal polyps (CRSwNP) and over half of patients with asthma show a type 2 inflammatory signature in sinonasal mucosa and/or lungs. Importantly, both chronic respiratory diseases are frequent comorbidities, ensuring alleviation of both upper and lower airway pathology by systemic biological therapy. Type 2‐targeting biologics such as anti‐IgE, anti‐IL4Rα, anti‐IL5, and anti‐IL5Rα have entered the market for selected pheno/endotypes of asthma patients and may soon also become available for CRSwNP patients. Given the high prevalence of chronic respiratory diseases and the high cost associated with biologics, patient selection is crucial in order to implement such therapies into chronic respiratory disease care pathways.

The European Forum for Research and Education in Allergy and Airway Diseases (EUFOREA) organized a multidisciplinary Expert Board Meeting to discuss the positioning of biologics into the care pathways for CRSwNP patients with and without comorbid asthma.

## 1 INTRODUCTION

Chronic rhinosinusitis (CRS) is a chronic inflammatory condition of the sinonasal cavities that affects 5%‐12% of the general population worldwide according to epidemiological studies.^1^, ^2^, ^3^, ^4^ The European Position Paper on Rhinosinusitis and Nasal Polyps (EPOS) defines CRS clinically based on symptoms supported by signs of mucosal inflammation found on imaging or with nasal endoscopy.^5^ Recently, the prevalence of clinically based CRS has shown to be between 3% and 6.4%.^6^, ^7^ CRS is classically divided into a phenotype with and without nasal polyps (CRSwNP and CRSsNP, respectively). Using patient questionnaires to measure the prevalence of CRSwNP yielded estimates of 2.1% (France) to 4.3% (Finland) in Europe and 1.1% in China.^8^ CRSwNP comprises a heterogeneous group of patients who differ with respect to coexisting asthma, allergy, NSAID‐exacerbated respiratory disease (N‐ERD),^9^ smoking, age of onset, and disease severity.^10^, ^11^, ^12^ Asthma affects 30%‐70% of the CRSwNP patients.^8^, ^10^, ^13^, ^14^ Conversely, the presence of nasal polyps is associated with the severity of asthma, regardless of smoking status ranging from 10%‐30% in mild asthma to 70%‐90% in severe asthma.^15^, ^16^ Both CRSwNP and asthma share common underlying pathophysiological mechanisms driving the disease (endotype), of which type 2 inflammation is the most prominent.^13^, ^17^, ^18^, ^19^ Type 2 inflammation is characterized by the presence of eosinophilic airway inflammation associated with type 2‐related cytokines (IL4, IL5, and/or IL13) and circulating and/or local IgE.^13^, ^20^

The management guideline in Europe for CRS, the European Position Paper on Rhinosinusitis and Nasal Polyps (EPOS), has been developed to provide physicians with comprehensive tables of levels of evidence and helpful management algorithms.^5^ In the United States, similar consensus statements have been published in 2016 by Orlandi et al.^21^

The cornerstone of the management of both CRSwNP and asthma consists of anti‐inflammatory treatment with local corticosteroids, aiming to achieve optimal disease control.^5^, ^21^, ^22^ When this is insufficient, short courses of oral corticosteroids are used (usually 30‐60 mg for 14 days, sometimes reducing over time).^23^, ^24^ Sinus surgery is the treatment option for CRSwNP patients in cases failing medical treatment.^25^, ^26^, ^27^ Recently, also more attention has been paid to the concept of “treatable traits.” Treatable traits have been postulated as a management concept which complements the traditional diagnostic labels such as CRSwNP or CRSsNP, thereby focusing on therapy targeted to a patient's individual disease‐associated characteristics.^28^, ^29^ Typical treatable traits in the upper airways can be smoking, allergy, occupation, and mucociliary clearance deficits.^30^

Biological therapies have entered the market for patients with asthma almost 15 years ago with anti‐IgE as first‐line therapy for patients with severe allergic asthma 31 and urticaria.^32^, ^33^, ^34^, ^35^ Recently, other monoclonal antibodies targeting type 2 inflammation 36 have been approved and are available now for patients with eosinophilic asthma,^37^, ^38^, ^39^, ^40^, ^41^ atopic dermatitis,^42^, ^43^ and urticaria.^36^, ^42^, ^43^, ^44^, ^45^, ^46^ A number of trials have been done with biological therapies for CRSwNP.^47^, ^48^, ^49^, ^50^ As these drugs enter the market, it necessitates the medical community to reflect on the positioning of these therapies in the current care pathways of the upper and lower airways.^51^, ^52^

The European Forum for Research and Education in Allergy and Airway Diseases organized a multidisciplinary Expert Board Meeting on November 29‐30, 2018, to develop proposals for the positioning of biologics into the care pathways for CRSwNP patients with or without asthma. Subsequently, a patient advisory board meeting was held to discuss the outcomes of the Expert Board Meeting.

## 2 SEVERITY AND THE BURDEN OF UNCONTROLLED DISEASE IN CRSwNP AND ASTHMA

CRSwNP has a severe impact on quality of life comparable to asthma 53, 54 and poses a significant burden on society.^54^, ^55^ In particular, the loss of sense of smell is a debilitating and often underappreciated component and can significantly impact one’s quality of life.^56^, ^57^

The terms “disease control” and “disease severity” cannot be used interchangeably. In CRSwNP, severity is defined by the impact of the symptoms on general quality of life and it can be measured with VAS and/or SNOT‐22.^58^ Uncontrolled disease in CRS is defined as persistent symptoms such as nasal blockage, mucopurulent rhinorrhea/postnasal drip, facial pain/headache, impaired sense of smell or sleep disturbance/fatigue, and/or diseased mucosa in the last 3 months or the need for long‐term antibiotics or systemic steroids in the last month.^5^, ^58^, ^59^ Few real‐life studies have evaluated the burden of uncontrolled disease following these criteria. A study performed at an academic referral center showed that at least 40% of CRS patients are uncontrolled despite maximal medical and surgical treatment 60.

The goal of CRS management is to achieve and maintain clinical control with minimal use of medication and associated side effects or surgical interventions. Additionally, the frequency of recurrence of nasal polyps and the need for systemic corticosteroids might be measures of disease control. In clinical practice, systemic corticosteroids are used more frequently and for longer periods than proposed in guidelines.^8^, ^60^ Real‐life studies are needed to determine the cumulative exposure to corticosteroids of patients with comorbid CRSwNP and asthma. The side effects of repeated use of systemic corticosteroids were also identified by the patient advisory board as a major concern.^61^

Symptomatic nasal polyp recurrence rates, defined as patients undergoing revision endoscopic sinus surgery, are reported to be 20% within a 5‐year period after surgery 62, 63 but may be as high as 50% on endoscopic examination.^62^

Type 2 disease is a strong predictor of recurrent disease with more than 50% of recurrences occurring in clusters with high eosinophilia.^62^, ^63^, ^64^, ^65^

The Global Initiative for Asthma (GINA) suggests assessing asthma severity retrospectively from the level of treatment required to control symptoms and exacerbations. Mild asthma is asthma that can be controlled with low‐dose inhaled corticosteroids. Severe asthma is defined as asthma that requires treatment with high‐dose inhaled corticosteroids (ICS) plus a second controller and/or systemic corticosteroids to maintain symptom control (after other causes of lack of control, that is, treatment adherence and inhalation technique have been addressed) or asthma that remains uncontrolled despite this (maximal) therapy.^66^

There is a clear correlation between control of upper and lower airways in patients with CRS and asthma and many patients with severe asthma have comorbid CRSwNP, which should be addressed to optimize asthma control.^67^, ^68^, ^69^ To conclude, the management of CRSwNP and asthma patients who are uncontrolled despite medical and often surgical intervention remains a challenge. However, in recent years, there has been significant innovation and expansion in the treatment armamentarium since the advent of biological therapies.

## 3 EFFICACY OF BIOLOGICAL TREATMENT FOR CRSwNP AND ASTHMA

Omalizumab was the first biological therapy that entered the market for patients with moderate‐to‐severe allergic asthma. It have been shown to improve disease control, reduce the number of asthma exacerbations, the need for oral corticosteroid, and rescue medication use.^31^, ^70^ In recent years, several other biologics (anti‐IL5, anti‐IL5R, and anti‐IL4Rα) have shown to be effective for the treatment of severe asthmatics with a type 2 inflammatory signature.^71^, ^72^ In most countries, biologics are indicated in moderate‐to‐severe asthma with insufficient level of control despite high dose of inhaled corticosteroids combined with at least one other asthma medication and where severe exacerbations and/or oral corticosteroid‐dependent asthma have been demonstrated.

The first proof‐of‐concept studies in CRSwNP using anti‐IgE, anti‐IL5, and anti‐IL4Rα strategies also showed promising results and have been summarized earlier.^50^, ^73^ Recent larger scale studies showed a moderate reduction in the need for surgery following treatment with anti‐IL5 in patients with CRSwNP.^48^ It was stated earlier that asthma is a frequent comorbidity in patients with CRSwNP. All trials with biologics in CRSwNP also showed a positive impact on the lower airways with significant changes in either AQLQ, ACQ‐5, or FEV_1 _in patients with comorbid asthma.^47^, ^48^, ^74^ Each of these biologics is tested in phase III clinical trials for CRSwNP patients with results to be expected in 2019. Preliminary data suggest a significant positive impact on quality of life, especially on the sense of smell and reduction in the need for surgery and systemic corticosteroid treatment.

## 4 INDICATIONS FOR BIOLOGICS

**Figure 1:**
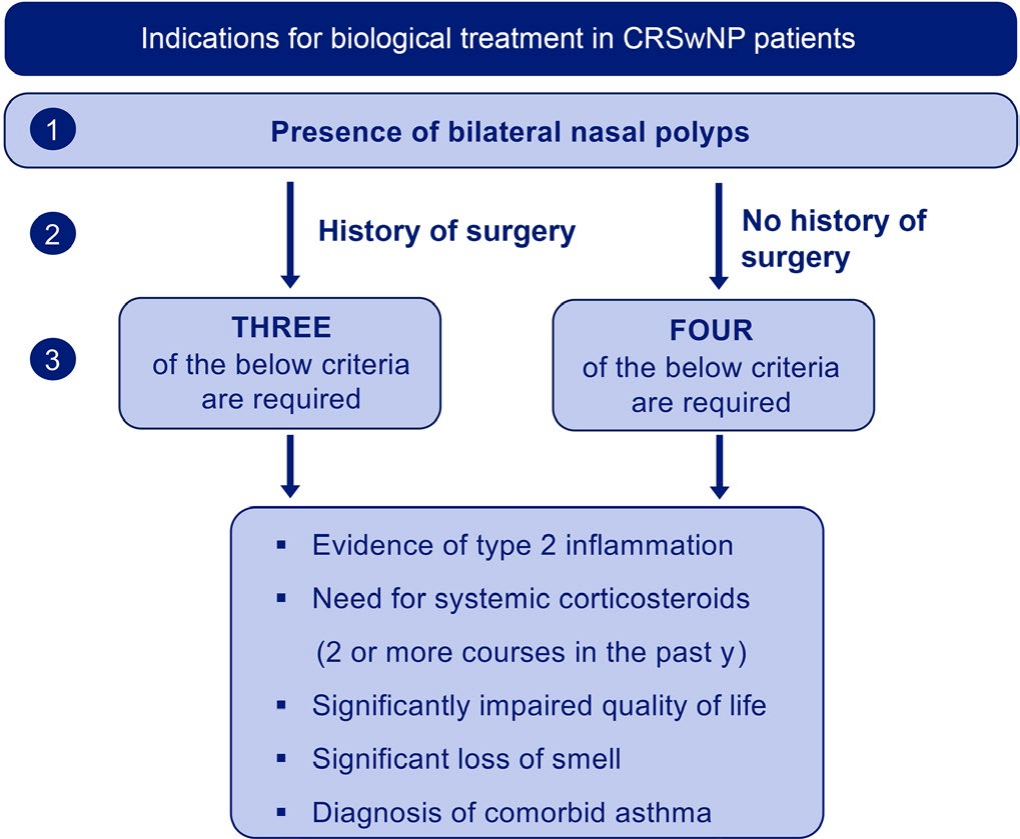
Indications for biological treatment in patients with CRSwNP: proposal of the multidisciplinary EUFOREA Expert Board Meeting

The high burden of uncontrolled disease, the recurrence of nasal polyps after sinus surgery, and the side effects associated with repeated courses of oral corticosteroids all underline the need for novel therapies. Given that biologics come with a high cost for the healthcare system, careful selection of patients is highly recommended. The EUFOREA expert team has put forward five criteria that are important in the decision to prescribe biologics in CRSwNP with prior sinus surgery (Figure 1):

Evidence of type 2 inflammation (biological biomarker)
Need for systemic corticosteroids in the past 2 years
Significant quality‐of‐life impairment
Significant loss of smell
Diagnosis of comorbid asthma

It was concluded that biologics are indicated in patients with bilateral nasal polyps who had undergone sinus surgery in the past and meet 3 of the above criteria.

There was an extensive discussion of whether there is a role for biologics in patients without previous sinus surgery. If these patients meet the criteria for severe asthma, they might fulfill the eligibility criteria to receive biological treatment by their pulmonologist.

In patients with severe CRSwNP and mild‐moderate asthma, the question as to whether biologics may become a valid alternative for sinus surgery is difficult to answer before the approval and introduction of biologics into the market. While most patients are keen to avoid surgery if possible, the effectiveness of biologics in preventing or reducing the need for surgery is yet to be established. The current evidence shows a significant but incomplete, relatively modest, reduction in polyp size, suggesting that a notable proportion of patients might still need surgery despite treatment with biologics.^37^, ^38^, ^39^ On the other hand, given that repeated surgeries cannot prevent recurrence in CRSwNP subjects with type 2 inflammation, and in line with the principles of precision medicine that patients also will share in decision making, it is likely that biologics will in time become an alternative for sinus surgery as currently performed.

To date, one study evaluated omalizumab vs sinus surgery in patients with grade 3 CRSwNP and asthma.^49^ It was concluded that omalizumab is equally effective in reducing SNOT‐22 at 16 weeks to sinus surgery. However, large‐scale studies are needed to confirm these findings in order to decide upon whether or not biologics could be a valid alternative to primary sinus surgery.

Therefore, it was concluded that patients who have never had sinus surgery need to meet at least 4 of the above criteria in order to be eligible for biological treatment.

Finally, indications not to initiate type 2 biological treatment were defined as follows:

CRSsNP and lack of signs of type 2 inflammation
Cystic fibrosis
Unilateral nasal polyps
Mucoceles
General contraindications for biological treatments, such as immunodeficiencies
Patient‐related factors such as noncompliance to therapy

## 5 DEFINING RESPONSE TO BIOLOGICS

**Figure 2:**
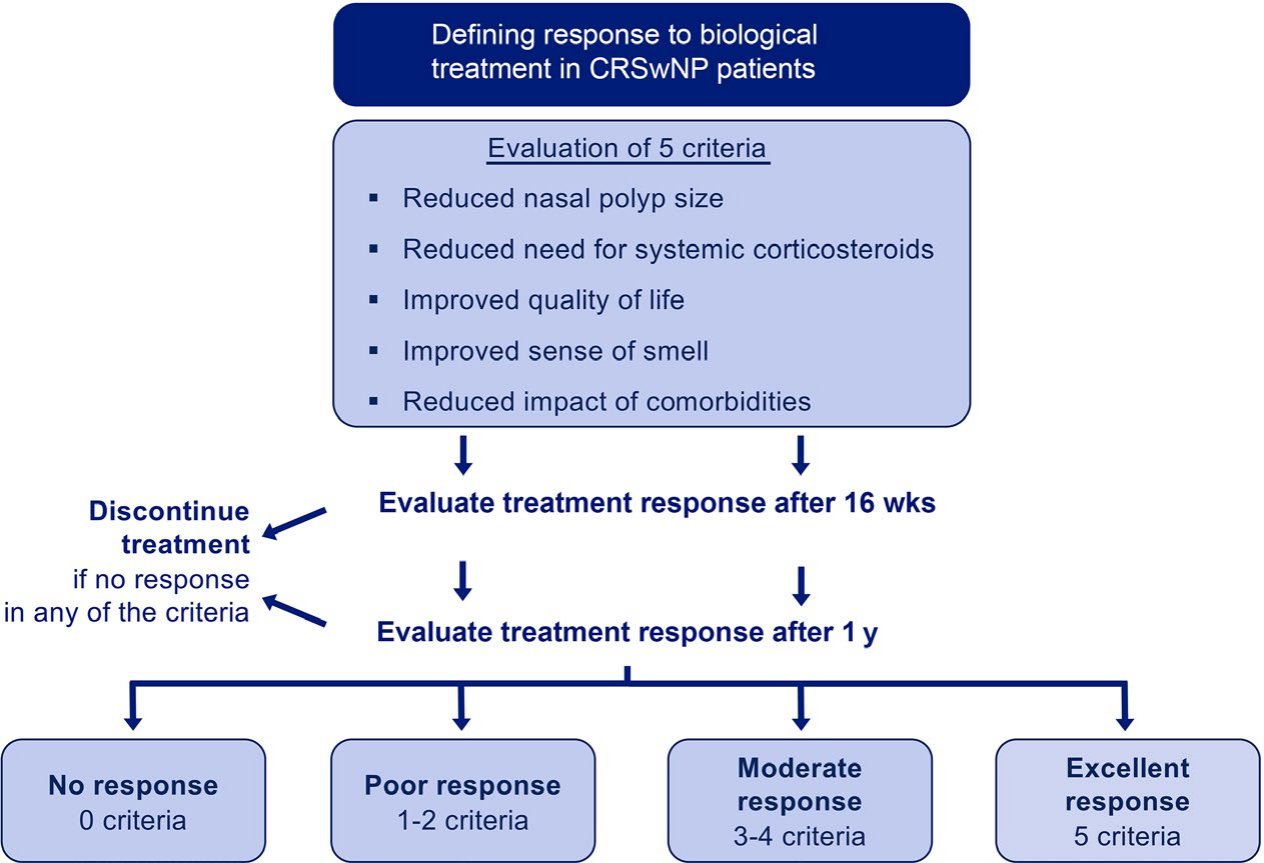
Response criteria for biological treatment in patients with CRSwNP: proposal of the multidisciplinary EUFOREA Expert Board Meeting

Despite significant efficacy of biologics on various clinical and patient‐reported outcome measures in the overall study population, considerable variability in the degree of response to such therapies is seen. These observations underpin the need to identify treatment responders as well as nonresponders. The following criteria were agreed by the expert team to define response to biological therapy after 1 year (Figure 2):

Reduced nasal polyp size
Reduced need for systemic corticosteroids
Improved quality of life
Improved sense of smell
Reduced impact of comorbidities

Three categories of response were defined as follows: poor (1‐2 criteria), good (3‐4 criteria), or excellent (5 criteria). It was proposed to assess the response to treatment after 16 weeks in order to decide upon continuation of the treatment (early stopping rule). The group felt that, ethically and clinically, an assessment point was required to avoid unnecessary continuation of a treatment which was not working and had chosen 16 weeks after discussion, but recognize that this will be validated/may change when further information becomes available from ongoing trials. It should be noted that real‐life studies are currently lacking to confirm the 16‐week early stopping time point.

## 6 POSITIONING OF BIOLOGICS IN THE CHRONIC RESPIRATORY DISEASE‐INTEGRATED CARE PATHWAY

New developments in understanding pathophysiology and treatment require new care pathways. Recently, integrated care pathways incorporating the different phenotypes and endotypes have been proposed.^75^, ^76^ Although, as we speak, biologics do not yet have an indication for CRSwNP, we can expect this to happen in the very near future.

Implementing integrated care pathways into daily clinical practice requires both collaboration between first, second, and third lines of care and across specialties (ENT, pulmonology, allergology). Patients pointed out during the advisory board meeting that awareness about CRS and nasal polyps and best‐practice management options are unsatisfactory. Thus, it is the patients’ perception that timely referral to a specialist is often delayed. Education of both patients and primary care physicians is thought to facilitate timely and accurate diagnosis of patients with CRSwNP and/or asthma. Because there are indications that early treatment of CRS may prevent asthma and further healthcare use,^77^ appropriate management at the right level of care may eventually prevent further development of disease and be highly cost‐effective. Patients with a high‐risk phenotype (asthma and N‐ERD) should be referred to specialist centers early in their disease to optimize multidisciplinary management.

Many patients will predominantly have upper or lower airway diseases. However, it is recommended that every patient with CRS gets at least one systematic evaluation for asthma and allergy preferably by a validated questionnaire and if at risk for asthma, spirometry to assess lung function; skin prick test or measurement of specific blood IgE; and measurement of blood eosinophil counts. Similarly, for patients with asthma it is recommended that every patient is evaluated for upper airway problems (rhinitis or CRS) and allergy preferably by a validated questionnaire; nasal endoscopy, skin prick test, or measurement of specific blood IgE; and measurement of blood eosinophil counts. However, a subgroup of patients with severe CRS and asthma may benefit from an intensified collaboration between ENT and pulmonologist and where appropriate allergologist.

Remarkably, only a few of the physicians in the Expert Board admitted to having a multidisciplinary outpatient clinic in place. Notwithstanding this, recommendations of the Board included the development of a multidisciplinary integrated care pathway and subsequent implementation in daily practice with systematic evaluation of both upper and lower airways at every visit; treatment adjustments with attention to the full unified airways; regular measurement of type 2 biomarkers; and monitoring of the use of systemic corticosteroids.

## 7 CONCLUSION AND UNMET RESEARCH NEEDS

A multidisciplinary EUFOREA Expert Board Meeting and patient advisory board came together under the auspices of the European Forum for Research and Education in Allergy and Airway Diseases. The participants formulated a proposal for the positioning of biologics into the care pathways for CRSwNP with or without asthma patients. Criteria for and against the use of biologics and response criteria were defined (Figures 1 and 2).

A series of unmet needs for future research were identified as follows:

Evaluation of biological treatment in CRSsNP with signs of type 2 inflammation
Biomarker research to identify responders to biological treatments
Evaluation of the disease‐modifying effect of biological treatments
Evaluation of required duration of treatment and discontinuation criteria
Protocols of long‐term treatment
Interplay between biologics and sinus surgery
Health‐economic research

## CONFLICT OF INTEREST

Dr. Diamant reports personal fees from AstraZeneca, personal fees from Sanofi‐Genzyme, during the conduct of the study; personal fees from Aquilon, personal fees from ALK, personal fees from Boehringer Ingelheim, personal fees from Gilead, personal fees from Hal Allergy, personal fees from MSD, outside the submitted work; and Apart from my academic affiliations I work at a phase I/II unit performing clinical studies for different biotech and pharma companies. Dr. Bachert reports personal fees from Sanofi, personal fees from GSK, personal fees from Novartis, personal fees from Astra‐Zeneca, during the conduct of the study. Dr. Bousquet reports personal fees from Chiesi, Cipla, Hikma, Menarini, Mundipharma, Mylan, Novartis, Sanofi‐Aventis, Takeda, Teva, Uriach, other from KYomed‐Innov, outside the submitted work. Dr Han reports to be consultant for Sanofi/Genzyme Regeneron and Astra‐Zeneca. Dr. Hellings reports grants and personal fees from Mylan, during the conduct of the study; personal fees from Sanofi, personal fees from Allergopharma, personal fees from Stallergenes, outside the submitted work. Dr. Hopkins reports personal fees from Advisory Board Participation ‐ Sanofi, personal fees from Advisory Board Participation – Glaxo Smith Kline, personal fees from Advisory Board Participation ‐ Optinose, personal fees from Advisory Board Participation – Smith and Nephew, outside the submitted work. Dr. J. Lee reports grants from Astra‐Zeneca, personal fees from Regeneron Healthcare Solutions, during the conduct of the study. Dr. Jankowski reports personal fees from sanofi regeneron, outside the submitted work. Dr. Joos reports grants and personal fees from AstraZeneca, personal fees from Eureca vzw, grants from Chiesi, grants and personal fees from GlaxoSmithKline, personal fees from Teva, outside the submitted work; all payments were done to his employer. Dr. S. Lee reports grants from Sanofi Regeneron, grants from Allakos Inc, grants from Astra Zeneca, other from Novartis, other from Sanofi Regeneron, outside the submitted work. Dr. Lund reports non‐financial support from GSK, grants from GSK, during the conduct of the study; personal fees from Abbott, personal fees from Kyorin, personal fees from MIMS, personal fees from MSD, personal fees from Elsevier Editor, outside the submitted work. Dr. Mullol reports personal fees and other from SANOFI‐GENZYME & REGENERON, NOVARTIS, and ALLAKOS; grants and personal fees from MYLAN Pharma and URIACH Group; and personal fees from ALK‐Abelló A/S, Menarini, and UCB, outside the submitted work. Dr. Heffler reports grants from AstraZeneca, grants from GSK, grants from Sanofi‐Genzyme, grants from Novartis, grants from Nestlè Purina, grants from Circassia, outside the submitted work. Dr Fokkens reports grants from Sanofi, grants from GSK, grants from Novartis, during the conduct of the study. Dr. Bjermer, Dr. Deneyer, Dr. Desrosiers, Dr. Knill, Dr. Mariën, Dr. Seys, Dr. Senior, Dr. Pugin hava nothing to disclose.

## AUTHOR CONTRIBUTIONS

All authors contributed to the discussion that was the base for this document and approved the content.

## References

[1] Hastan D , Fokkens WJ , Bachert C , et al. Chronic rhinosinusitis in Europe–an underestimated disease. A GA2LEN study. Allergy. 2011;66(9):1216‐1223. 21605125 10.1111/j.1398-9995.2011.02646.x

[2] Hirsch AG , Stewart WF , Sundaresan AS , et al. Nasal and sinus symptoms and chronic rhinosinusitis in a population‐based sample. Allergy. 2017;72(2):274‐281. 27590749 10.1111/all.13042 PMC5497579

[3] Ostovar A , Fokkens WJ , Vahdat K , Raeisi A , Mallahzadeh A , Farrokhi S . Epidemiology of chronic rhinosinusitis in Bushehr, southwestern region of Iran: a GA2LEN study. Rhinology. 2018;57(1):43‐48. 10.4193/Rhin18.061 30033451

[4] Shi JB , Fu QL , Zhang H , et al. Epidemiology of chronic rhinosinusitis: results from a cross‐sectional survey in seven Chinese cities. Allergy. 2015;70(5):533‐539. 25631304 10.1111/all.12577 PMC4409092

[5] Fokkens WJ , Lund VJ , Mullol J et al. European Position Paper on Rhinosinusitis and Nasal Polyps 2012. Rhinol Suppl. 2012;23:3 p preceding table of contents, 1‐298. 22764607

[6] Dietz de Loos D , Lourijsen ES , Wildeman M , et al. Prevalence of chronic rhinosinusitis in the general population based on sinus radiology and symptomatology. J Allergy Clin Immunol. 2019;143(3):1207‐1214. 30578880 10.1016/j.jaci.2018.12.986

[7] Tomassen P , Newson RB , Hoffmans R , et al. Reliability of EP3OS symptom criteria and nasal endoscopy in the assessment of chronic rhinosinusitis–a GA(2) LEN study. Allergy. 2011;66(4):556‐561. 21083566 10.1111/j.1398-9995.2010.02503.x

[8] Khan A , Vandeplas G , Huynh T , et al. The global allergy and asthma European network (GALEN rhinosinusitis cohort: a large European cross‐sectional study of chronic rhinosinusitis patients with and without nasal polyps. Rhinology. 2019;57(1):32‐42. 29911211 10.4193/Rhin17.255

[9] Kowalski ML , Agache I , Bavbek S et al. Diagnosis and management of NSAID‐exacerbated respiratory disease (N‐ERD)‐a EAACI position paper. Allergy. 2019;74(1):28‐39. 30216468 10.1111/all.13599

[10] Philpott CM , Erskine S , Hopkins C , et al. Prevalence of asthma, aspirin sensitivity and allergy in chronic rhinosinusitis: data from the UK National Chronic Rhinosinusitis Epidemiology Study. Respir Res. 2018;19(1):129. 29945606 10.1186/s12931-018-0823-y PMC6020303

[11] Wu D , Bleier BS , Li L , et al. Clinical phenotypes of nasal polyps and comorbid asthma based on cluster analysis of disease history. J Allergy Clin Immunol Pract. 2018;6(4):1297‐305.e1. 29100865 10.1016/j.jaip.2017.09.020

[12] Liao B , Liu J‐X , Li Z‐Y , et al. Multidimensional endotypes of chronic rhinosinusitis and their association with treatment outcomes. Allergy 2018;73(7):1459‐1469. 29331025 10.1111/all.13411 PMC6019131

[13] Tomassen P , Vandeplas G , Van Zele T , et al. Inflammatory endotypes of chronic rhinosinusitis based on cluster analysis of biomarkers. J Allergy Clin Immunol. 2016;137(5):1449‐1456.e4. 26949058 10.1016/j.jaci.2015.12.1324

[14] Langdon C , Mullol J . Nasal polyps in patients with asthma: prevalence, impact, and management challenges. J Asthma Allergy. 2016;9:45‐53. 27042129 10.2147/JAA.S86251 PMC4798207

[15] Lin DC , Chandra RK , Tan BK , et al. Association between severity of asthma and degree of chronic rhinosinusitis. Am J Rhinol Allergy. 2011;25(4):205‐208. 21819754 10.2500/ajra.2011.25.3613 PMC3390198

[16] Shaw DE , Sousa AR , Fowler SJ , et al. Clinical and inflammatory characteristics of the European U‐BIOPRED adult severe asthma cohort. Eur Respir J. 2015;46(5):1308‐1321. 26357963 10.1183/13993003.00779-2015

[17] Zhang Y , Derycke L , Holtappels G , et al. Th2 cytokines orchestrate the secretion of MUC5AC and MUC5B in IL‐5‐positive chronic rhinosinusitis with nasal polyps. Allergy. 2019;74(1):131‐140. 29802623 10.1111/all.13489

[18] De Greve G , Hellings PW , Fokkens WJ , Pugin B , Steelant B , Seys SF . Endotype‐driven treatment in chronic upper airway diseases. Clin Transl Allergy. 2017;7:22. 28706720 10.1186/s13601-017-0157-8 PMC5506670

[19] Seys SF , Scheers H , Van den Brande P , et al. Cluster analysis of sputum cytokine‐high profiles reveals diversity in T(h)2‐high asthma patients. Respir Res 2017;18(1):39. 28231834 10.1186/s12931-017-0524-y PMC5324270

[20] Green RH , Brightling CE , Woltmann G , Parker D , Wardlaw AJ , Pavord ID . Analysis of induced sputum in adults with asthma: identification of subgroup with isolated sputum neutrophilia and poor response to inhaled corticosteroids. Thorax 2002;57(10):875‐879. 12324674 10.1136/thorax.57.10.875 PMC1746199

[21] Orlandi RR , Kingdom TT , Hwang PH , et al. International consensus statement on allergy and rhinology: rhinosinusitis. Int Forum Allergy Rhinol. 2016;6(Suppl 1):S22‐209. 26889651 10.1002/alr.21695

[22] Pedersen SE , Bateman ED , Boulet L‐P et al. e. 2018 GINA report, global strategy for Asthma management and prevention. https://ginasthma.org/wp-content/uploads/2018/04/wms-GINA-2018-report-tracked_v1.3.pdf;2018://ginasthma.org/wp-content/uploads/2018/04/wms-GINA-2018-report-tracked_v1.3.pdf;2018 10.1183/09031936.00138707 18166595

[23] Pundir V , Pundir J , Lancaster G , et al. Role of corticosteroids in functional endoscopic Sinus surgery–a systematic review and meta‐analysis. Rhinology. 2016;54(1):3‐19. 26970247 10.4193/Rhin15.079

[24] Voorham J , Xu X , Price DB , et al. Healthcare resource utilization and costs associated with incremental systemic corticosteroid exposure in asthma. Allergy. 2019;74(2):273‐283. 29987879 10.1111/all.13556 PMC6587525

[25] Rudmik L , Soler ZM , Hopkins C , et al. Defining appropriateness criteria for endoscopic sinus surgery during management of uncomplicated adult chronic rhinosinusitis: a RAND/UCLA appropriateness study. Rhinology. 2016;54(2):117‐128. 26934470 10.4193/Rhino16.023

[26] Hopkins C , Surda P , Bast F , Hettige R , Walker A , Hellings PW . Prevention of chronic rhinosinusitis. Rhinology. 2018;56(4):307‐315. 30052695 10.4193/Rhin17.027

[27] Kilty SJ , Lasso A , Mfuna‐Endam L , Desrosiers MY . Case‐control study of endoscopic polypectomy in clinic (EPIC) versus endoscopic sinus surgery for chronic rhinosinusitis with polyps. Rhinology. 2018;56(2):155‐157. 29306958 10.4193/Rhin17.115

[28] Yii A , Tay TR , Choo XN , Koh M , Tee A , Wang DY . Precision medicine in united airways disease: A "treatable traits" approach. Allergy. 2018;73(10):1964‐1978. 29869791 10.1111/all.13496

[29] Tay TR , Hew M . Comorbid, "treatable traits" in difficult asthma: Current evidence and clinical evaluation. Allergy. 2018;73(7):1369‐1382. 29178130 10.1111/all.13370

[30] Fokkens WJ , Reitsma S . Proposal for an algorithm on the management of chronic rhinosinusitis. Allergy. 2019;74 (in press). 10.1111/all.13797 30916791

[31] Busse WW , Anti‐immunoglobulin E . (omalizumab) therapy in allergic asthma. Am J Respir Crit Care Med. 2001;164(8 Pt 2):S12‐S17. 11704612 10.1164/ajrccm.164.supplement_1.2103026

[32] Asero R . Efficacy of omalizumab 150 mg/month as a maintenance dose in patients with severe chronic spontaneous urticaria showing a prompt and complete response to the drug. Allergy. 2018;73(11):2242‐2244. 29989179 10.1111/all.13549

[33] Cugno M , Asero R , Ferrucci S , et al. Elevated IgE to tissue factor and thyroglobulin are abated by omalizumab in chronic spontaneous urticaria. Allergy. 2018;73(12):2408‐2411. 30076634 10.1111/all.13587

[34] Ertas R , Ozyurt K , Atasoy M , Hawro T , Maurer M . The clinical response to omalizumab in chronic spontaneous urticaria patients is linked to and predicted by IgE levels and their change. Allergy. 2018;73(3):705‐712. 29083482 10.1111/all.13345

[35] Kaplan AP , Gimenez‐Arnau AM , Saini SS . Mechanisms of action that contribute to efficacy of omalizumab in chronic spontaneous urticaria. Allergy. 2017;72(4):519‐533. 27861988 10.1111/all.13083 PMC5915348

[36] Hassani M , Koenderman L . Immunological and hematological effects of IL‐5(Ralpha) targeted therapy: an overview. Allergy. 2018;73(10):1979‐1988. 29611207 10.1111/all.13451 PMC6220846

[37] Castro M , Corren J , Pavord ID , et al. Dupilumab efficacy and safety in moderate‐to‐severe uncontrolled asthma. N Engl J Med. 2018;378(26):2486‐2496. 29782217 10.1056/NEJMoa1804092

[38] Farne HA , Wilson A , Powell C , Bax L , Milan SJ . Anti‐IL5 therapies for asthma. Cochrane Database Syst Rev. 2017;9:CD010834. 28933516 10.1002/14651858.CD010834.pub3 PMC6483800

[39] Rabe KF , Nair P , Brusselle G , et al. Efficacy and safety of dupilumab in glucocorticoid‐dependent severe asthma. N Engl J Med. 2018;378(26):2475‐2485. 29782224 10.1056/NEJMoa1804093

[40] Diamant Z , Vijverberg SJ , Agache I , et al. Much ado about Biologicals: highlights of the master class on biologicals, Prague, 2018. Allergy. 2019;74(4):837‐840. 30506938 10.1111/all.13688

[41] Diamant Z , Vijverberg S , Alving K , et al. Towards clinically applicable biomarkers for asthma ‐ An EAACI position paper. Allergy. 2019. (in press). 10.1111/all.13806 30953574

[42] Simpson EL , Bieber T , Guttman‐Yassky E , et al. Two phase 3 trials of Dupilumab versus placebo in atopic dermatitis. N Engl J Med. 2016;375(24):2335‐2348. 27690741 10.1056/NEJMoa1610020

[43] Blauvelt A , de Bruin‐Weller M , Gooderham M , et al. Long‐term management of moderate‐to‐severe atopic dermatitis with dupilumab and concomitant topical corticosteroids (LIBERTY AD CHRONOS): a 1‐year, randomised, double‐blinded, placebo‐controlled, phase 3 trial. Lancet. 2017;389(10086):2287‐2303. 28478972 10.1016/S0140-6736(17)31191-1

[44] Weller K , Ohanyan T , Hawro T , et al. Total IgE levels are linked to the response of chronic spontaneous urticaria patients to omalizumab. Allergy. 2018;73(12):2406‐2408. 30076605 10.1111/all.13586

[45] Staubach P , Metz M , Chapman‐Rothe N , et al. Omalizumab rapidly improves angioedema‐related quality of life in adult patients with chronic spontaneous urticaria: X‐ACT study data. Allergy. 2018;73(3):576‐584. 29058822 10.1111/all.13339 PMC5836932

[46] Spekhorst LS , van den Reek J , Knulst AC , Rockmann H . Determinants of omalizumab drug survival in a long‐term daily practice cohort of patients with chronic urticaria. Allergy 2018. (in press). 10.1111/all.13714 30593687

[47] Bachert C , Mannent L , Naclerio RM , et al. Effect of subcutaneous dupilumab on nasal polyp burden in patients with chronic sinusitis and nasal polyposis: a randomized clinical trial. JAMA 2016;315(5):469‐479. 26836729 10.1001/jama.2015.19330

[48] Bachert C , Sousa AR , Lund VJ , et al. Reduced need for surgery in severe nasal polyposis with mepolizumab: randomized trial. J Allergy Clin Immunol. 2017;140(4):1024‐1031.e14. 28687232 10.1016/j.jaci.2017.05.044

[49] Bidder T , Sahota J , Rennie C , Lund VJ , Robinson DS , Kariyawasam HH . Omalizumab treats chronic rhinosinusitis with nasal polyps and asthma together‐a real life study. Rhinology. 2018;56(1):42‐45. 29288573 10.4193/Rhino17.139

[50] Tsetsos N , Goudakos JK , Daskalakis D , Konstantinidis I , Markou K . Monoclonal antibodies for the treatment of chronic rhinosinusitis with nasal polyposis: a systematic review. Rhinology. 2018;56(1):11‐21. 29396960 10.4193/Rhino17.156

[51] Fokkens WJ , Bachert C , Bernal‐Sprekelsen M , et al. Rhinology future debates, an EUFOREA report. Rhinology. 2017;55(4):298‐304. 29166426 10.4193/Rhin17.221

[52] Hellings PW , Akdis CA , Bachert C , et al. EUFOREA rhinology research forum 2016: report of the brainstorming sessions on needs and priorities in rhinitis and rhinosinusitis. Rhinology. 2017;55(3):202‐210. 28501885 10.4193/Rhin17.028

[53] Dudvarski Z , Djukic V , Janosevic L , Tomanovic N , Soldatovic I . Influence of asthma on quality of life and clinical characteristics of patients with nasal polyposis. Eur Arch Otorhinolaryngology 2013;270(4):1379‐1383. 10.1007/s00405-012-2242-x 23135235

[54] Sahlstrand‐Johnson P , Hopkins C , Ohlsson B , Ahlner‐Elmqvist M . The effect of endoscopic sinus surgery on quality of life and absenteeism in patients with chronic rhinosinuitis ‐ a multi‐centre study. Rhinology. 2017;55(3):251‐261. 28624844 10.4193/Rhin16.126

[55] Smith KA , Orlandi RR , Rudmik L . Cost of adult chronic rhinosinusitis: a systematic review. Laryngoscope. 2015;125(7):1547‐1556. 25640115 10.1002/lary.25180

[56] Croy I , Nordin S , Hummel T . Olfactory disorders and quality of life–an updated review. Chem Senses 2014;39(3):185‐194. 24429163 10.1093/chemse/bjt072

[57] Hummel T , Whitcroft KL , Andrews P , et al. Position paper on olfactory dysfunction. Rhinol Suppl. 2017;54:1-30. 29528615 10.4193/Rhino16.248

[58] Toma S , Hopkins C . Stratification of SNOT‐22 scores into mild, moderate or severe and relationship with other subjective instruments. Rhinology. 2016;54(2):129‐133. 27017484 10.4193/Rhino15.072

[59] Hellings PW , Fokkens WJ , Akdis C , et al. Uncontrolled allergic rhinitis and chronic rhinosinusitis: where do we stand today? Allergy. 2013;68(1):1‐7. 10.1111/all.12040 23025484

[60] van der Veen J , Seys SF , Timmermans M , et al. Real‐life study showing uncontrolled rhinosinusitis after sinus surgery in a tertiary referral centre. Allergy. 2017;72(2):282‐290. 27392210 10.1111/all.12983 PMC5248621

[61] Winblad L , Larsen CG , Hakansson K , Abrahamsen B , von Buchwald C . The risk of osteoporosis in oral steroid treatment for nasal polyposis: a systematic review. Rhinology. 2017;55(3):195‐201. 28492609 10.4193/Rhin15.367

[62] DeConde AS , Mace JC , Levy JM , Rudmik L , Alt JA , Smith TL . Prevalence of polyp recurrence after endoscopic sinus surgery for chronic rhinosinusitis with nasal polyposis. Laryngoscope. 2017;127(3):550‐555. 27859303 10.1002/lary.26391 PMC5321782

[63] Hopkins C , Slack R , Lund V , Brown P , Copley L , Browne J . Long‐term outcomes from the English national comparative audit of surgery for nasal polyposis and chronic rhinosinusitis. Laryngoscope. 2009;119(12):2459‐2465. 19780032 10.1002/lary.20653

[64] Vlaminck S , Vauterin T , Hellings PW , et al. The importance of local eosinophilia in the surgical outcome of chronic rhinosinusitis: a 3‐year prospective observational study. Am J Rhinol Allergy. 2014;28(3):260‐264. 24980239 10.2500/ajra.2014.28.4024

[65] Wei B , Liu F , Zhang J , et al. Multivariate analysis of inflammatory endotypes in recurrent nasal polyposis in a Chinese population. Rhinology. 2018;56(3):216‐226. 29785413 10.4193/Rhin17.240

[66] Chung KF , Wenzel SE , Brozek JL , et al. International ERS/ATS guidelines on definition, evaluation and treatment of severe asthma. Eur Respir J. 2014;43(2):343‐373. 24337046 10.1183/09031936.00202013

[67] Schlosser RJ , Smith TL , Mace J , Soler ZM . Asthma quality of life and control after sinus surgery in patients with chronic rhinosinusitis. Allergy. 2017;72(3):483‐491. 27638398 10.1111/all.13048 PMC5315592

[68] Phillips KM , Bergmark RW , Hoehle LP , Caradonna DS , Gray ST , Sedaghat AR . Chronic rhinosinusitis exacerbations are differentially associated with lost productivity based on asthma status. Rhinology. 2018;56(4):323‐329. 30042985 10.4193/Rhin18.033

[69] Phillips KM , Hoehle LP , Bergmark RW , et al. Chronic rhinosinusitis severity is associated with need for asthma‐related systemic corticosteroids. Rhinology. 2017;55(3):211‐217. 28647751 10.4193/Rhin17.029

[70] Solèr M , Matz J , Townley R , et al. The anti‐IgE antibody omalizumab reduces exacerbations and steroid requirement in allergic asthmatics. Eur Respir J. 2001;18(2):254‐261. 11529281 10.1183/09031936.01.00092101

[71] Magnan A , Bourdin A , Prazma CM , et al. Treatment response with mepolizumab in severe eosinophilic asthma patients with previous omalizumab treatment. Allergy. 2016;71(9):1335‐1344. 27087007 10.1111/all.12914 PMC5089585

[72] Pepper AN , Renz H , Casale TB , Garn H . Biologic therapy and novel molecular targets of severe asthma. J Allergy Clin Immunol Pract. 2017;5(4):909‐916. 28689841 10.1016/j.jaip.2017.04.038

[73] Bachert C , Zhang L , Gevaert P . Current and future treatment options for adult chronic rhinosinusitis: focus on nasal polyposis. J Allergy Clin Immunol. 2015;136(6):1431‐1440. 26654192 10.1016/j.jaci.2015.10.010

[74] Gevaert P , Calus L , Van Zele T , et al. Omalizumab is effective in allergic and nonallergic patients with nasal polyps and asthma. J Allergy Clin Immunol. 2013;131(1):110‐6.e1. 23021878 10.1016/j.jaci.2012.07.047

[75] Bachert C , Zhang N , Hellings PW , Bousquet J . Endotype‐driven care pathways in patients with chronic rhinosinusitis. J Allergy Clin Immunol. 2018;141(5):1543‐1551. 29731100 10.1016/j.jaci.2018.03.004

[76] Hellings PW , Fokkens WJ , Bachert C , et al. Positioning the principles of precision medicine in care pathways for allergic rhinitis and chronic rhinosinusitis ‐ A EUFOREA‐ARIA‐EPOS‐AIRWAYS ICP statement. Allergy. 2017;72(9):1297‐1305. 28306159 10.1111/all.13162

[77] Hopkins C , Rimmer J , Lund VJ . Does time to endoscopic sinus surgery impact outcomes in chronic rhinosinusitis? Prospective findings from the national comparative audit of surgery for nasal polyposis and chronic rhinosinusitis. Rhinology. 2015;53(1):10‐17. 25756072 10.4193/Rhino13.217

